# Assessing multiple sclerosis-related quality of life among Iranian patients using the MSQOL-54 tool: a cross-sectional study

**DOI:** 10.1186/s12883-021-02357-8

**Published:** 2021-08-31

**Authors:** Shadi Ziaie, Niloofar Namazi, Golnaz Afzal, Saghar Barati, Rezvaneh Mohebbi, Mahshad Mir, Hadi Esmaily, Gholamhossein Mehralian

**Affiliations:** 1 Department of Clinical Pharmacy, School of Pharmacy, Shahid Beheshti University of Medical Sciences, Tehran, Iran; 2 Department of Clinical Pharmacy, School of Pharmacy, Shahid Sadoughi University of Medical Sciences, Yazd, Iran; 3 Department of Pharmaco-economics and Pharma Management, School of Pharmacy, Shahid Beheshti University of Medical sciences, Tehran, Iran

**Keywords:** Dietary supplement, Sclerosis, multiple, Health-related quality of life, Multiple sclerosis quality of Life-54, Iran

## Abstract

**Background:**

Multiple sclerosis (MS) is a chronic autoimmune disease and is one of the most costly medical conditions that imposed families with catastrophic health expenditures. There is an increasing trend in using alternative medicines including, dietary supplements, herbs, vitamins, and minerals. To date, the association between dietary as well as herbal supplements and QoL in MS patients is under researched; thus, this study aimed to assess the association between the self-reported supplement used and QoL between MS patients.

**Methods:**

This cross-sectional study was conducted on patients with MS referring to Shahid Kazemi Pharmacy, based in the city of Tehran, Iran, as a national pharmacy providing specialized pharmaceutical products and pharmaceutical care to patients. The Multiple Sclerosis Quality of Life-54 (MSQoL-54) tools was performed to evaluate MS patients QoL.

**Results:**

A total number of 382 patients with MS participated in this study. They include 89 (23.3%) men and 293 (76.7%) women, aged 40 ± 10.9 years old. The overall score of the MSQoL-54 questionnaire was 41.58 out of 100. Physical health composite (PHC) and mental health composite (MHC) were 69.60 and 62.99 from 100, respectively. This study revealed that 76.4% of patients used at least one vitamin daily; 92.4% of patients do not receive any herbal product. Vitamin D is the most widely used supplement, followed by calcium, while vitamin C is the least consumed. No correlation was observed regarding supplement use and overall QoL, PHC, or MHC. There were no significant differences between QoL’s dimensions score in patients who used supplements. The results showed that increasing the number of supplements used did not relate to overall QoL, PHC, or MHC. In addition, there was not any correlation between the duration used of supplements and QoL’s dimensions score in MS patients (*p*-value> 0.05).

**Conclusions:**

The dietary supplement appears to be popular among MS patients. The study results showed that the number of supplementations and their long-term use in patients with MS were not associated with higher QoL. Similarly, the herbal supplements have failed to improve QoL.

## Background

Multiple Sclerosis (MS) is known as the major autoimmune demyelinating disease of the central nervous system (CNS) and even one of the main causes of disabilities, high healthcare costs, and mortality in young adults [1–3]. The reported cases of MS in 2016 have shown 2,221,188 patients living with MS across the world [4]. Iran is also a country with a high prevalence rate of MS in the Middle East. In 2019, the incidence of MS among Iranians had been similarly reported to range from 7 to 148.1/100,000 [5]. Patients with MS often reported the role limitations due to a wide variety of MS symptoms such as spasms, ataxia, vertigo, fatigue, sexual dysfunction, pain, vision loss, paresis, urinary or fecal sphincter dysfunction, and tremor [6, 7]. Besides, MS negatively impacts cognitive and psychological functions, impacting their health-related quality of life (QoL) [8–10].

Research has shown that patients with MS have lower QoL than the general population and suffer from other chronic neurological disorders like Parkinson’s disease and epilepsy [11].

Knowledge of which factors could influence QoL in patients with MS is critical, enhancing health authorities and policymakers in clinical decision-making and assisting them in choosing the most appropriate interventions [12–14]. It has largely focused on the dietary supplement as one of the possible environmental factors with QoL’s physical and mental dimensions such as MS symptomatology and psychological status [15]. Some studies showed that the pattern of dietary supplements used among MS patients is increasing [16, 17]; up to 70% of patients with MS have tried one or more complementary and alternative medicine treatments for their disease [18]. Therefore, MS patients are frequently interested in using supplements to improve their QoL [16, 19]. The evidence clearly shows that supplying accessories would increasingly cost patients [20]. while it is found, there is no always a significant positive relationship between costs of MS and patient’s QoL [21–23].

The finding of several clinical studies in MS patients has demonstrated that some dietary supplements could decrease the severity of MS symptoms and thus improve QoL [15, 24–28]. At the same time, there are few studies examining associations between self-reported supplement use and MS patient’s QoL [29]. On the other hand, there is no effective clinical indication authorized by food and drug agencies for applying dietary supplementation against MS symptomatology and enhancement of QoL [15, 30]. Furthermore, there is very limited research evaluating the relationship between the real-world supplement used and dimensions of QoL such as physical and MHC in MS patients. In the other word, the role of minerals, trace elements, antioxidants, and vitamins, which has received increased attention in the past decade among MS patients on QoL, is unclear. However, it is essential to evaluate the QoL as a significant clinical outcome in patients with MS and assess the role of the dietary supplement used in the real world on QoL among patients with MS [15, 31, 32].

Several measurement tools of QoL have been identified [13]. However, MS patients’ QoL is often measured by the Multiple Sclerosis Quality of Life-54 (MSQoL-54) questionnaire, which has been typically practiced in clinical studies in recent years [12, 33]. In this sense, we used the MSQoL-54 as the most common and standardized disease-specific questionnaire to assess QoL in patients with MS [34] based on the generic SF-36 QoL instrument [35]. Therefore, the objectives of the present study were to assess QoL, pattern dietary supplement usage among patients living with MS, and identify if the type of supplements used could be related to improve QoL.

## Methods

### Study design

This cross-sectional study was conducted from February 2019 to March 2020 at Shahid Kazemi Pharmacy in Tehran, Iran, as a national pharmacy providing specialized pharmaceutical care to patients with MS. The Ethics Committee also approved this study of Shahid Beheshti University of Medical Sciences, Tehran, Iran, with the registry code of IR.SBMU.PHARMACY.REC.1398.240.

### Study population and data collection

The patients affected with MS referring to the pharmacy concerned were invited to participate in this study. After scrutinizing their prescriptions, additional questions were further raised to ensure that the patients had been diagnosed with MS. To be included in the study, the cases needed to be older than 18 years and at least 6 months of MS diagnosis. Written informed consent was also given to the patients before their inclusion, and they were allowed to withdraw from the study whenever they desired. The sample size was calculated using Cochran’s formula. According to the statistics released by the Iranian MS Society, the number of patients with MS in Iran was 68,192 cases. In this formula, the confidence interval (CI) of 1.96 Z-score was 95%, and the *p*-value, as the ratio of the attribute in the society, was equal to 0.5. Besides, the margin of error (D) in this study was 0.05. The sample size was further estimated to be 382 individuals.

### Questionnaire development

The validated Persian version of the MSQoL-54 questionnaire was applied to collect the required data, whose acceptable reliability and construct validity had been already confirmed [36]. Notably, the MSQoL-54 is known as a health-related self-report questionnaire containing 54 items, categorized into 12 sub-scales: physical health (10 items), role limitations-physical (4 items), emotional well-being (8 items), pain (3 items), energy (5 items), health perceptions (5 items), social function (3 items), cognitive function (4 items), health distress (4 items), sexual function (5 items), change in health (1 item), and overall QoL (2 items). There was also one item under the theme of overall QoL, related to patients’ views about overall assessment of their own QoL, labeled as “self-score” in this study.

Additionally, two composite scores, namely, physical health composite (PHC) and mental health composite (MHC), were measured by adding some sub-scales accordingly. The PHC includes eight sub-scales of physical function, health perceptions, energy/fatigue, role limitations-physical, pain, sexual function, social function, and health distress and the MHC is made up of five sub-scales: health distress, overall QoL, emotional well-being, role limitations-emotional, and cognitive function. The composite scores can be calculated by transforming the item scores to zero to 100 scales, with zero representing the worst health status and 100 indicating the best health status.

With respect to demographic data, characteristics such as age, gender, marital status, levels of education, job, income, physical activity, comorbidity (heart disease, diabetes, hypertension, hypothyroid, hyperthyroid, cancer, stroke, and depression), MS subtype, MS medication, mode of administration, duration of MS disease, and the concomitant use of an anxiolytic agent or an antidepressant were asked (Table 1). The authors developed a structured self-administered questionnaire to measure the *usage patterns* of vitamins, minerals, herbals, and another dietary supplements with detailed information about the type, the number of supplements taken, and the continuity of taking supplements. Physical activity was measured using the SQUASH, a valid and reliable questionnaire to determine daily physical activity based on an average week in the past month in MS patients [37, 38]. The SQUASH was also comprised of items on commuting activities, leisure-time and sports activities, household activities, and activities at work and school. Based on the reported efforts in the SQUASH questionnaire, the patients were divided into three classes according to their physical activity level as follows: low, moderate, and high [39].

**Table 1:** Demographic and clinical characteristics of patients (*n* = 382)

Variable	N (%) or Mean ± SD
**Age**, years (mean ± SD)	40 ± 10.9
**Gender** (male)	89 (23.3%)
**Marital status**	
Single	154 (40.3%)
Married	228 (59.7%)
**level of education**	
Undergraduate	27(7.1%)
Diploma	102(26.7%)
Associate degree	15(3.9%)
Bachelor	152(39.8%)
Master	64(16.8%)
Professional doctoral degree	22(5.8%)
**Job**	
Unemployed	28 (7.3%)
Freelance	134 (35.1%)
Government	16 (4.2%)
Housewife	168 (44%)
Student	13 (3.4%)
Retired	23 (6%)
**Income**	
No income	214 (56%)
1–5 million toman/month	81 (21.2%)
5–10 million toman/month	62 (16.2%)
> 10 million toman/month	25 (6.5%)
**Physical activity**	
Low	246 (64.4%)
Moderate	88 (23%)
High	48 (12.6%)
**Comorbidity**	
Heart disease	6 (1.6%)
Diabetes	7 (1.8%)
Hypertension	15 (3.9%)
Hypothyroid	20 (5.2%)
**Hyperthyroid**	2 (0.5%)
Cancer	6 (1.6%)
Stroke	1 (0.3%)
Depression	19 (5%)
Duration of MS* disease, months	111 ± 78
**MS subtype**	
RRMS*	354 (92.7%)
PMS*	28 (7.3%)
**Mode of MS medication administration**	
Oral	254 (66.5%)
Injection	128 (33.5%)
**Anti-depressant drugs**	
Tricyclic antidepressants	1%
Selective serotonin reuptake inhibitors	1.7%
Serotonin-norepinephrine reuptake inhibitor	1.5%
Atypical	0.5%
**Antianxiety drugs**	
anticonvulsant	2.4%
benzodiazepines	1.3%
Nonbenzodiazepines	0.5%
**Supplements**	
Yes	305 (79.8%)
No	77 (20.2%)
Number of daily supplements used	1 ± 1.3
Supplement used duration, months	79.2 ± 105

*MS* Multiple sclerosis, *RRMS* relapsing-remitting Multiple sclerosis, *PMS* Progressive multiple sclerosis

### Statistical analyses

Descriptive statistics were used for assessing all demographic data and the parameters related to patients’ profiles. The Kolmogorov–Smirnov test was used to evaluate the distribution of the data. For comparisons, an independent sample t-test was used. Correlations between the physical, mental health composite and overall QoL scores with the other parameters such as; the number of supplement usage daily and supplement duration were evaluated using Pearson’s correlation test. The level of significance was set at 0.05. Statistical analyses were done using SPSS 23.0 software (SPSS Inc., Chicago, IL, USA).

## Results

Over 15 months, a total number of 382 MS patients agreed to participate in the study, including 89 (23.3%) men and 293 (76.7%) women with the mean age of 40 ± 10.9 years. The mean disease duration was also 111 ± 78 months ranged from 6 to 480 months. Based on the clinical subtype of MS, 354 (92.7%) of patients were identified as RRMS, and 254 (66.5%) received oral disease-modifying therapies. Of the 382 MS patients enrolled, 305 (79.8%) used dietary supplements regularly daily. The mean duration of supplement usage was 79.2 ± 105. In this study, the concomitant use of antidepressant and anxiolytic drugs was just 4.7 and 4.2%, respectively.

All other demographic characteristics of the participants are depicted in Table 1.

**Fig. 1:**
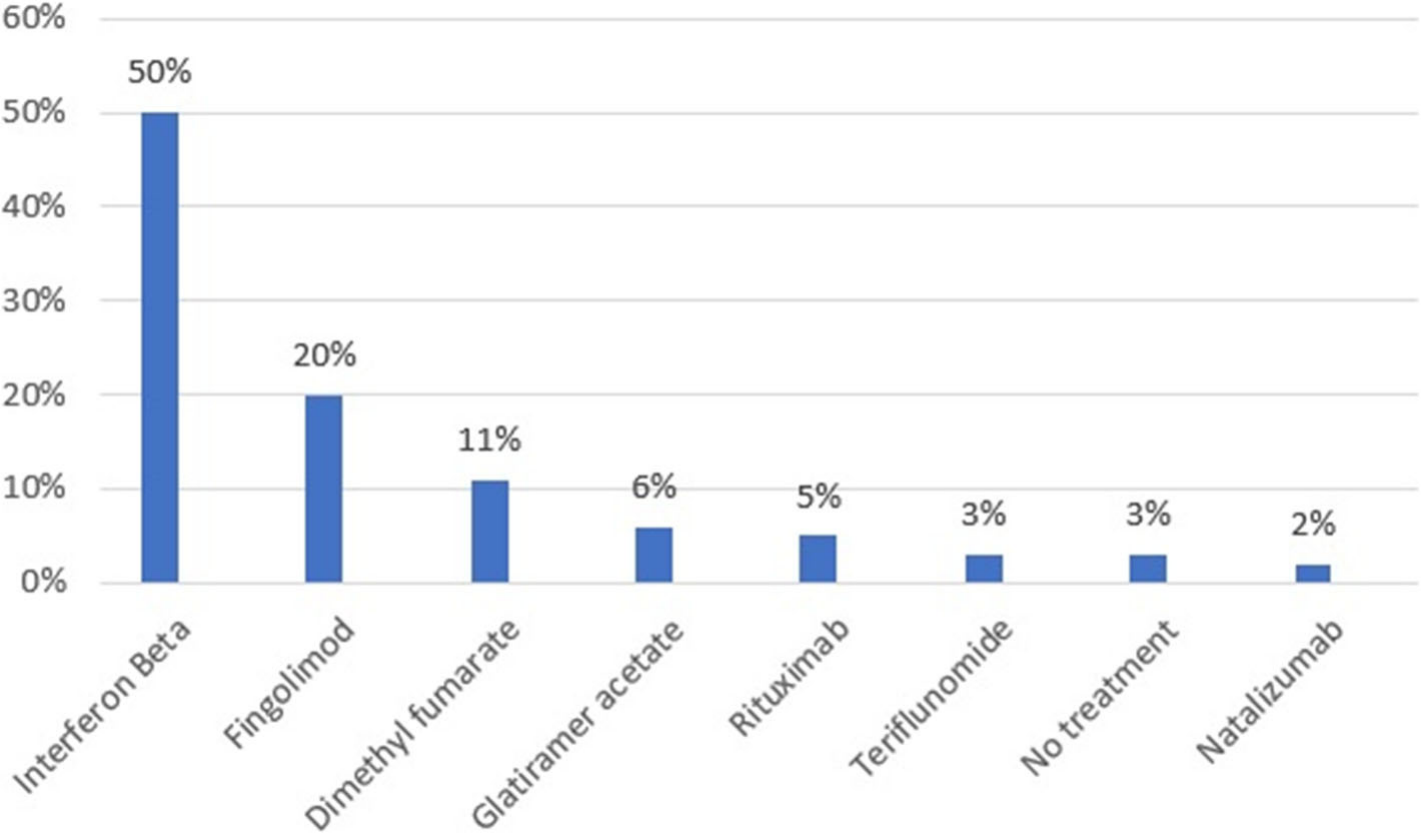
Frequency distribution of medication among patients with multiple sclerosis (N: 382)

The frequency of MS medications taken by the patients indicates in (Fig. 1). Interferon beta was the most commonly prescribed medication to treat MS.

In addition, Table 2 presents that the overall score of the MSQoL-54 questionnaire among the patients with MS was 41.58 ± 12.69, and that was 69.60 ± 18.38 and 62.99 ± 22.74 respectively for the PHC and MHC.

**Table 2:** The mean and standard deviation of MSQoL-54 dimensions among patients with multiple sclerosis

Variable	Mean score	SD
Physical Health Composite	69.60	18.38
Mental Health Composite	62.99	22.74
Overall QoL score	41.58	12.69

*QoL* quality of life, *SD* standard deviation

**Fig. 2:**
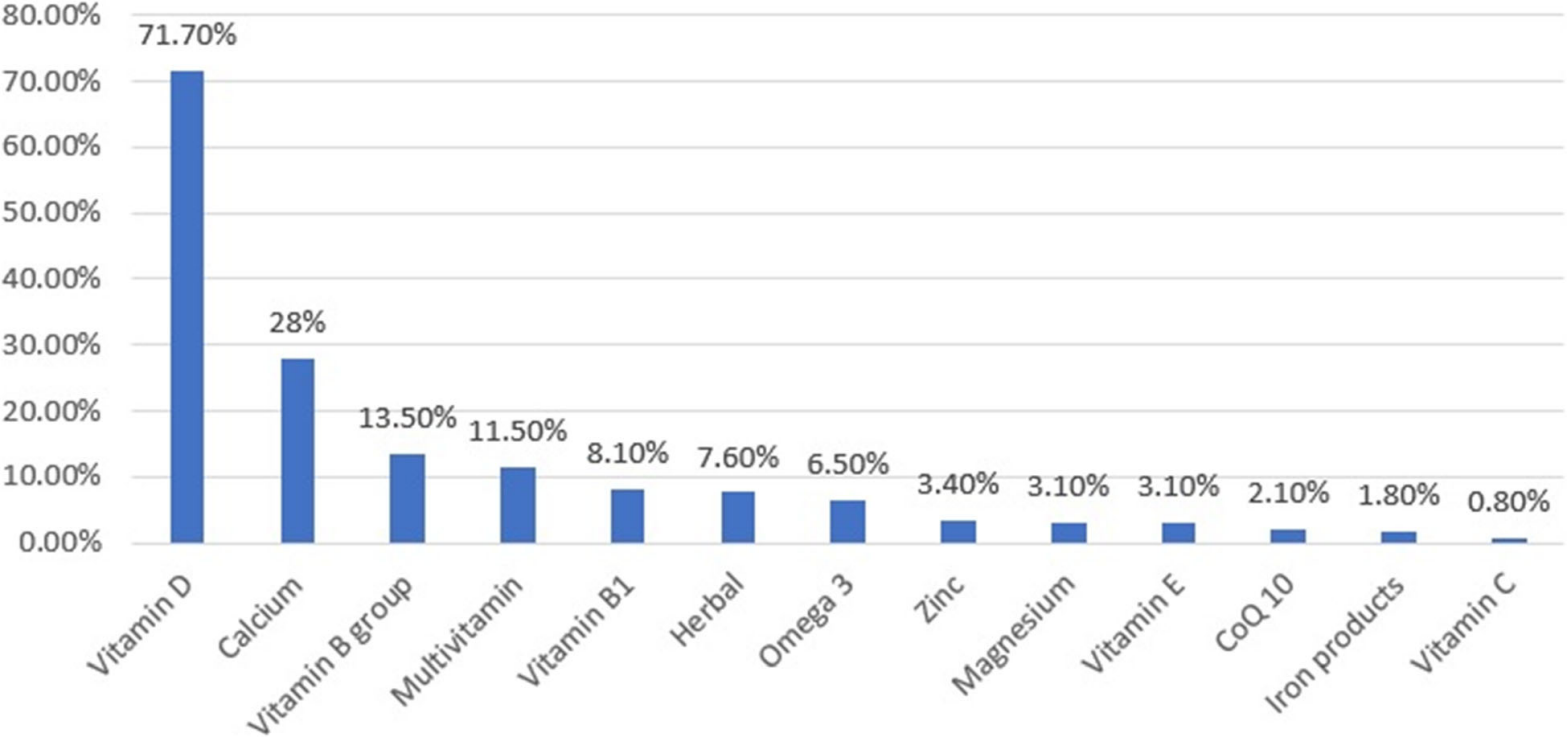
Frequency distribution of the dietary and herbal supplements among patients with multiple sclerosis (N: 382)

The results of this study reveal that 76.4% of patients used at least one vitamin daily (Table 3). Figure 2 shows that only 7.6% of the patients received the herbal supplements. Vitamin D is the most widely used supplement, followed by calcium, while vitamin C is the least consumed.

**Table 3:** Number of vitamins usage daily by patients with multiple sclerosis (N:382)

Number of vitamins usage daily	N (%)
None	90 (23.6)
One	203 (53.1)
Two	60 (15.7)
Three	23 (6)
Four	6 (1.6)

Moreover, given the normality of data, parametric analysis was applied for all studies. Table 4 shows the comparison of QoL dimensions between 382 MS patients who received dietary and herbal supplements or not. Patients who take calcium had less physical health status. Besides, there was a significant inverse relationship between the consumption of herbal and vitamin C supplements and patients’ overall QoL. In contrast, those receiving iron products were associated with more PHC sore.

**Table 4:** Comparison of QoL dimensions between 382 patients with multiple sclerosis who received the dietary and herbal supplement

Treatment	Physical health (Mean ± SD)	PV*	Mental health (Mean ± SD)	PV*	Overall QoL (Mean ± SD)	PV*
Vitamin D3	Yes: 114 ± 8	0.330	Yes: 63 ± 22	0.271	Yes: 41 ± 12	0.490
	No: 108 ± 3		No: 60 ± 24		No: 40 ± 13	
Calcium	Yes: 103 ± 57	0.038	Yes: 59 ± 22	0.052	Yes: 39 ± 12	0.076
	No: 116 ± 58		No: 64 ± 22		No: 42 ± 12	
Vitamin B group	Yes: 108 ± 52	0.557	Yes: 62 ± 19	0.815	Yes: 42 ± 10	0.317
	No: 113 ± 59		No: 63 ± 23		No: 41 ± 13	
Multi vitamin	Yes: 115 ± 57	0.746	Yes: 64 ± 21	0.651	Yes: 43 ± 12	0.343
	No: 112 ± 58		No: 62 ± 22		No: 41 ± 12	
Vitamin B1	Yes: 109 ± 51	0.678	Yes: 62 ± 18	0.826	Yes: 43 ± 9	0.250
	No: 113 ± 59		No: 63 ± 23		No: 41 ± 12	
Herbal	Yes: 116 ± 57	0.852	Yes: 61 ± 20	0.767	Yes: 35 ± 14	0.040
	No: 113 ± 59		No: 63 ± 23		No: 42 ± 12	
Omega-3	Yes: 92 ± 55	0.069	Yes: 59 ± 23	0.394	Yes: 40 ± 11	0.725
	No: 114 ± 58		No: 63 ± 22		No: 41 ± 12	
Zinc	Yes: 111 ± 55	0.932	Yes: 62 ± 19	0.951	Yes: 38 ± 14	0.404
	No: 113 ± 58		No: 63 ± 22		No: 41 ± 12	
Magnesium	Yes: 115 ± 55	0.871	Yes: 62 ± 19	0.908	Yes: 43 ± 12	0.519
	No: 112 ± 58		No: 63 ± 22		No: 41 ± 12	
Vitamin E	Yes: 129 ± 54	0.302	Yes: 68 ± 18	0.374	Yes: 37 ± 15	0.278
	No: 112 ± 58		No: 62 ± 22		No: 41 ± 12	
Co Q10	Yes: 78 ± 50	0.091	Yes: 54 ± 25	0.259	Yes: 37 ± 11	0.302
	No: 113 ± 58		No: 63 ± 22		No: 41 ± 12	
Iron product	Yes: 168 ± 13	< 0.01	Yes: 77 ± 16	0.097	Yes: 41 ± 15	0.950
	No: 112 ± 58		No: 62 ± 22		No: 41 ± 12	
Vitamin C	Yes: 111 ± 48	0.948	Yes: 55 ± 10	0.542	Yes: 21 ± 10	0.006
	No: 113 ± 58		No: 63 ± 22		No: 41 ± 12	

*P < 0.05 indicates significant difference

Table 5 shows that an increase in the number of supplements used did not relate to overall QoL, PHC, or MHC. No correlation was further observed regarding the supplement use duration in terms of overall QoL, PHC, or MHC (*p*-value> 0.05).

**Table 5:** Correlation of number and duration of supplements usage with QoL dimentions score

Variable	PHC		MHC		Overall OoL	
	Sig*	r**	Sig*	r**	Sig*	r**
Number of daily supplement	0.309	−0.052	0.512	−0.034	0.376	−0.045
Supplement used duration	0.422	0.041	0.069	0.093	0.192	0.067

*P < 0.05 indicates significant difference

## Discussion

This study aimed to determine the self-reported prevalence and types of dietary and herbal supplement use in MS patients and assess the role of dietary supplementation on the QoL of MS patients. The results confirm that a large proportion (79.8%) of MS patients in this study use supplements. The most common supplements reported were vitamin D, followed by calcium and vitamin B groups. This study suggests that daily supplementation with herbal, vitamin C, iron products, and calcium can significantly associate with a different part of QoL dimensions. Consistent with prior research [40–42], our study showed an increasing trend in using supplementary and alternative medicines among MS patients. For example, O’Connor et al. found that the frequency of dietary supplement use was about 82.1% among MS patients [16]. Another study was conducted in the USA reported that 44.8% of patients with MS used vitamins [19]. We found that 76.4% of MS patients used at least one vitamin daily. Additionally, our results showed that just 7.6% of MS patients used herbal supplements, ranging from 26 to 80% in other studies [40, 42, 43].

Differences among these studies may be due to different geographic areas, varied supplements studied, various categorizing patterns, and cultural differences [16, 19, 41]. The most commonly used dietary supplement by MS patients in our study was vitamin D. These results are similar to trends found in the published studies [15, 44]. The clinical effect of vitamin D on MS patient’s QoL is contradictory [45, 46]. Our findings favored those who did not confirm the significant impact of vitamin D on improved QoL.

It can be explained by decreased serological and metabolic responsiveness to vitamin D supplementation by patients with MS. To address this concern, higher doses of vitamin D might be needed to have clinically relevant effects [47–49]. Similarly, such controversial results were found about herbal supplements [50, 51]. Our results showed that patients who received the herbal supplement had lower overall QoL. It could be argued that patients with lower QoL had felt disappointed with conventional therapies to control MS; they might show a higher willingness to use herbal supplements as complementary and alternative medicines.

Additionally, some herbal supplements may cause adverse reactions and interfere with MS conventional treatment; for example, herbal supplement containing echinacea may interfere with corticosteroids immunosuppressants [52–54]. Therefore, such an interaction may result from more demyelination and axonal loss, which leads to a vast number of troublesome MS symptoms and reduce QoL [55, 56].

The present study results showed that receiving more supplements could not increase overall QoL, PHC, and MHC. We also found no correlation between the duration of supplement use and QoL. In contrast, some clinical studies reported that the frequent use of nutritional supplements such as vitamin B12 and folic acid has a significant, positive effect on QoL of MS patients [28, 57]. Therefore, more studies are needed to evaluate this effect using real-world studies.

On the other hand, Ernstsson O and et al*.* were also observed that medication and dietary supplements were the main cost drivers for MS patients [20]. Hence, given the increasing evidence for the significant reverse associations between costs related MS disease and patient’s QoL [21–23], no specific supplement was recommended to improve QoL in MS patients. With regard to the influence of calcium and iron on MS patient’s QoL, our results showed that patients who received calcium had lower PHC, whereas who receive iron product had higher PHC. As it is well documented that certain micronutrient such as calcium and iron could control the progression of the MS disease, leading to improved QoL [44, 58], the reverse effect of calcium in this study might be related to this notion that the patients had already less physical activity status, it is consumed to postpone disease progression.

The findings were also consistent with earlier studies reporting that QoL was poor in patients living with MS [59, 60]. As mentioned by Ruth Ann Marrie et al., underdiagnosed and undertreated depression had been one of the fundamental reasons for reduced QoL in MS [61]. This study also reported only a few patients using antidepressant or anxiolytic drugs to keep their QoL at higher levels. For this reason, it is recommended that due to the progressive and disabling nature of MS and the negative effects of depression on QoL, the patients should be continuously evaluated and appropriate pharmacotherapy is given if needed.

This study had some limitations that should be taken into account. First, MSQOL-54 has not been specifically validated for evaluating correlation between MS QoL and supplement usag. Second, self-report bias would be potentially another limitation across all studies using such a method. Coming together these limitations, it is of grate practical to continue research aimed at the assessment of other aspects of using dietary and herbal supplement by MS patients in terms of the economic status, social support, or rehabilitation needs.

## Conclusions

Given the findings of this study, the dietary supplements appear to be popular among MS patients. Taking more supplements and their long-term usage were not posivitely associated with higher QoL. Similarly, the use of herbal supplements has failed to improve QoL. It is recommended to perform further self-reporting studies to evaluate the effectiveness of dietary and herbal supplements in enhancing MS-related QoL.

## Data Availability

The datasets used and/or analyzed during the current study are available from the corresponding author on reasonable request.

## Abbreviations

MS: Multiple sclerosis
MHC: Mental Health Composite
MSQOL-54: Multiple sclerosis quality of life-54 questionnaire
PHC: Physical Health Composite
QOL: Quality of life
